# New data on African health professionals abroad

**DOI:** 10.1186/1478-4491-6-1

**Published:** 2008-01-10

**Authors:** Michael A Clemens, Gunilla Pettersson

**Affiliations:** 1 Center for Global Development, 1776 Massachusetts Ave. NW, Suite 301, Washington, DC 20036, USA; 2 Public Policy Institute, Georgetown University, 3520 Prospect St. NW, 4th Fl., Washington, DC 20007, USA; 3 Department of Economics, University of Sussex, Brighton, BN1 9RE, UK

## Abstract

**Background:**

The migration of doctors and nurses from Africa to developed countries has raised fears of an African medical brain drain. But empirical research on the causes and effects of the phenomenon has been hampered by a lack of systematic data on the extent of African health workers' international movements.

**Methods:**

We use destination-country census data to estimate the number of African-born doctors and professional nurses working abroad in a developed country circa 2000, and compare this to the stocks of these workers in each country of origin.

**Results:**

Approximately 65,000 African-born physicians and 70,000 African-born professional nurses were working overseas in a developed country in the year 2000. This represents about one fifth of African-born physicians in the world, and about one tenth of African-born professional nurses. The fraction of health professionals abroad varies enormously across African countries, from 1% to over 70% according to the occupation and country.

**Conclusion:**

These numbers are the first standardized, systematic, occupation-specific measure of skilled professionals working in developed countries and born in a large number of developing countries.

## Background

Policy and academic debate – often impassioned – on health professional migration from developing countries has frequently advanced beyond systematic knowledge of the extent of the phenomenon. In South Africa, epicenter of the HIV catastrophe, the health minister recently claimed that "if there is a single major threat to our overall health effort, it is the continued outward migration of key health professionals, particularly nurses" [1]. After the UK National Health Service ended its active recruitment of staff from Sub-Saharan Africa in 2001, the British Medical Association (BMA) praised this "strong moral lead," adding that "[i]t is now essential that other developed countries ... make a similar commitment to address the issue" [2]. BMA Chairman of Council James Johnson flatly declared that "the rape of the poorest countries must stop" [3].

In academic circles, Harvard's Sabina Alkire and Lincoln Chen urge that developed countries' migration policy "should adopt 'medical exceptionalism' based on moral and ethical grounds" [4]. Devesh Kapur and John McHale caution against "poaching" health workers from developing countries and claim that the case is "obvious" for "restraint" in the recruitment of doctors and nurses [5]. Philip Martin, Manolo Abella, and Christiane Kuptsch assert that South Africa is "suffering" from a "brain drain" of doctors and nurses and decry a fiscal impact over $1 billion [6].

Some of the above statements were carefully researched using available information. But they were based (through no fault of the authors) on the available incomplete and problematic measures of the extent of health professional migration because systematic data on international flows of African health workers have simply been absent. Untested hypotheses abound.

The simple reason for this is that no agency collects standardized data on international flows of people disaggregated by occupation. Each scholar who approaches the issue of African health professional migration is thus obliged to collect data anew. Amy Hagopian et al. use professional association data to count the number of African-trained physicians from nine sending countries practicing in two receiving countries (the US and Canada) [7]. Fitzhugh Mullan reports the number of physicians trained in eight African countries (and in Sub-Saharan Africa in the aggregate) practicing in four Anglophone destination countries [8]. The World Health Organization lists data on African-trained doctors and nurses working in seven or eight destination countries, covering 10 sending countries for doctors and 19 for nurses [9]. In a more ambitious effort, Docquier and Bhargava report the number of 'African' physicians from each of *all *the African sending countries practicing in 16 receiving countries each year from 1991 to 2004 – where 'African' is defined differently according to each receiving country [10].

### Limitations of other investigations

Each of these valuable datasets, while useful for certain research questions, has important characteristics that limit its application to other questions. First, three of these studies count only physicians; they omit nurses and all other types of health professionals, who are of great importance to African health systems and who constitute the majority of the health professional diaspora from Sub-Saharan Africa. Second, the studies of Hagopian et al., Mullan, and WHO only report a limited number of sending and receiving countries, giving a poor idea of total flows – especially for non-Anglophone African countries.

Third, the Hagopian et al., Mullan, and WHO data focus exclusively – and Docquier and Bhargava primarily – on African-*trained *physicians as the principal measure of physicians' departure from Africa. This approach would lead to decent statistics for a study of, say, the fiscal consequences of physician emigration; the vast majority of African-trained doctors are trained with public funds. (It would be problematic even for this purpose, however, since a portion of African doctors trained abroad do so using scholarships funded by their home governments.) A statistic measuring diaspora size based on country of birth would be a poor indicator indeed of the fiscal consequences of emigration. But a narrow focus on country of training would not be appropriate for other studies – such as an investigation of the effects of physician emigration on health system staffing, health care availability, or health outcomes in the countries of origin. We explain below.

To see this, note that 12 of the 53 countries in Africa (and 11 of 48 Sub-Saharan countries) do not have a medical school accredited by the Foundation for Advancement of International Medical Education and Research (FAIMER) [11]. A medical degree from a FAIMER-accredited school is a prerequisite to licensure in major receiving countries such as the United States [12], and related but effectively similar restrictions apply in Australia and Canada. This means that, properly measured, an indicator of physician 'drain' based strictly on country of training would *define *about a quarter of Sub-Saharan Africa to have lost *zero *physicians to emigration. It is certain, however, that physicians would have left most or all of those countries to some degree at some point, with possible consequences for staffing, the availability of care or health outcomes. For related reasons, a country-of-training based measure would artificially define nurse emigration from most Francophone African countries to be extremely small, since French law currently mandates that only graduates from French nursing schools may practice as professional nurses in France. Home-trained nurses who leave must therefore train again, in France, in order to appear in the data as practicing nurses in France – so they become foreign-trained nurses. Beyond this, a country-of-training measure for either doctors or nurses would give an odd accounting even for countries that do have accredited schools but many of whose nationals nevertheless train in other African countries. For instance: Doctors in the UK who were born in Zambia and Zimbabwe, but who trained in South Africa, would contribute exclusively to the South African 'brain drain' – a classification that might be sensible for some research questions, but not others.

Fourth, the Docquier and Bhargava data take the very problematic step of mixing different and highly conflicting measures of what constitutes an African physician abroad. 'African' physicians are counted in some destination countries by their country of birth (e.g. Belgium), in others by their country of citizenship (e.g. Portugal), and in others by their country of training (e.g. France). This fact renders the meaning of the blended database extremely vague. To give one example, the French Ordre National des Médecins reports that in 1999 there were 238 physicians in France who were citizens of Sub-Saharan African countries, but the French census of 1999 reports 4,203 physicians in France who were born in Sub-Saharan Africa – a difference of 1,766%! To make another comparison, in 2001 the Canadian Medical Association reports 190 physicians in Canada trained in Egypt, but the Canadian census of the same year shows 750 Egyptian-*born *physicians working in Canada (a 395% difference). Such discrepancies are the rule, not the exception. Differences of this magnitude suggest that mixing these different classifications can destroy the ability of the resulting number to measure anything at all. In empirical studies of emigrants and diasporas it is imperative to choose a single definition and retain it.

Fifth, there are limits to the coverage of the Docquier and Bhargava data in time and space. They report panel data on 14 years of annual flows of physicians out of Africa, but these are calculated based on 14 years of annual stock data for only five of the 16 destination countries they study. In the other 11 receiving countries the flows are interpolated from three or fewer annual observations (in 10 of them, 2 or fewer observations). For the large majority of the receiving countries, then, the annual flows are broad interpolations. The result is a database that is a blend of cross section and time series, with an unknown degree of measurement error in either dimension. Finally, the dataset omits destination countries that are very important for certain African sending countries, destinations like Spain and South Africa.

The present study seeks to create a systematic, standardized snapshot of the stock of African-born physicians and professional nurses living and working in developed countries. It improves on earlier work by including professional nurses; by maintaining a single, consistent definition of 'African'; by including all the major destination countries; by covering every African sending country; and by providing information on country of birth rather than country of training, a more useful measure for certain research questions.

Crucially, the numbers presented here do *not *represent the number of Africans who became health professionals in Africa and subsequently departed Africa. They only and exclusively represent the numbers of African people who have two traits: 1) they work outside Africa and 2) they work as health professionals. Only a subset of these people became health professionals in Africa and subsequently moved. If the latter is the population of interest, however, then for the reasons discussed above, simply counting up health professionals outside Africa who were trained in Africa (as is often done in the literature) is not an adequate measure either. As we have explained, this would severely undercount physician 'drain' from one quarter of sub-Saharan African countries, as well as nurse 'drain' from all of Francophone Africa. The simple fact is that no single, one-size-fits-all measure of health professional 'drain' exists. A statistic describing the size of the African health professional diaspora based on country of birth is not a measure of 'drain', but captures interesting information relevant to research questions about 'drain'; the same is true of a statistic focusing on country of training.

## Methods

In late 2005 we contacted the census bureaus of the nine most important destination countries for African health professional emigrants to obtain estimates of the number of African-born doctors and nurses living in each destination country at the time of the most recent census.

### What is an 'African' health professional?

There is, of course, no single statistic that captures the extent of "African health worker emigration". One can interpret each component of the phrase in multiple ways. Is an "African" someone resident in Africa, someone born in Africa, someone whose ancestors for several generations were born in Africa, someone trained in Africa, or someone who holds African citizenship? Does "Africa" include North Africa and all of South Africa? Is a "health worker" someone who was trained as such or someone who currently works in the health sector? How long must one stay outside the country for that movement to be "emigration"?

This database takes one of many possible valid stances on these questions. Here, we classify "Africans" by country of birth; we include the entire African continent; we count as doctors and nurses only those currently employed as doctors and nurses; we include only developed countries as destinations; and we count those who were residing in the receiving countries on a sufficiently permanent basis circa 2000 to be included in that country's most recent census.

All previous databases and this one share limitation that they are based on census or professional society data and thus record each individual's occupation as the job that the person performs currently. An African trained as a nurse who now works abroad outside the health sector is therefore not counted. But to the extent that the tendency for emigrant health professionals to leave the health sector does not differ markedly by country of origin, even numbers that do not account for this phenomenon still give a good indicator of relative emigration across sending countries. In other words, a certain number of emigrated Senegalese nurses are not counted because they no longer work as nurses. But there is no a priori reason to think this tendency stronger (nor thus that undercounting is greater) for Senegalese nurses than for Malawian nurses. An additional reason the data are informative despite the absence of those who leave the health profession in the destination countries is that some research questions will focus primarily on those who remain in the health field. A key question for policy research is whether or not developed countries are luring specifically health workers from poor countries to fill developed-country positions, and the incentive systems they create to do so only function to the extent that the immigrants remain in health care.

The case of Mozambique aptly illustrates the sensitivity of data like these to different assumptions. The Mozambique Medical Association estimates, in a personal communication to the authors, that only around 5% of Mozambican physicians work abroad. Destination-country census data show that about 75% of people born in Mozambique who now work as physicians do not live in Mozambique. The main cause of this disparity is the fact that many of those physicians are of European ancestry and departed in the mass exodus of Portuguese colonists around independence in 1975. But it is not at all obvious that counting whites results in a poor measure of human capital loss. In South Africa white health professionals today play an important role in educating a new generation of black health professionals. It is true that Mozambican-born physicians in the white colonist class were providing most of their health care to urban elites in the colonial era rather than to rural blacks, but the same could be said of many black physicians in today's independent African states. We take country of birth as a useful measure of "African-ness" though we recognize it is not germane to all research questions. To restate this point, 1) white African colonial doctors have made and do make some contribution to health conditions for black Africans, and 2) many black African doctors have only a limited impact on health conditions for the mass of black Africans, for example because many focus their practices on elites who live in urban areas. It is not at all clear, therefore that a measure of the African health professional diaspora restricted only to certain ethnic groups is a superior measure for all or even most research questions.

### Nine destination countries proxy for the world

We also assume that we have a good estimate of how many African health professionals live outside each sending country simply by counting how many live in the nine most important destination countries. Those countries are the United Kingdom, United States, France, Australia, Canada, Portugal, Belgium, Spain, and South Africa. In choosing this list we sought a balance between coverage and the time and expense of additional data collection.

The primary reason that we take these countries as sufficient for most purposes to capture health professional emigration from Africa is that the first eight receiving countries alone account for 94.2% of all African-born, university-educated people residing in any OECD country in 2000. Our experience comparing the migration patterns of African health professionals to those of other types of well-educated migrants suggests that the proportion of total African health professional emigrants is similar to this value. We add a ninth country, South Africa, because we take it to be the most important non-OECD receiving country for African health professionals.

It is of course possible that another non-OECD country, such as Saudi Arabia, is important for some countries, or that health professionals differ greatly in their migration patterns from other skilled professionals. But survey data from African health professionals considering emigration suggest that neither of these is the case. Between 2001 and 2002, Magda Awases et al. of the World Health Organization interviewed 2,382 doctors, nurses, and other health professionals in six African countries [13]. Each person declaring an intention to emigrate was asked his or her favored destination. The fraction of these in each country who gave one of our nine destination countries was 89.3% in Cameroon, 91.8% in Senegal, and 94.6% in South Africa. A small percentage of respondents in Zimbabwe mentioned Botswana and New Zealand as destinations but the vast majority mentioned one of our nine receiving countries. Respondents from Ghana and Uganda did not mention any countries outside Africa besides the US and UK, and these two destinations plus South Africa accounted for the vast majority of favored destinations in both cases.

Martha Johanna Oosthuizen surveyed in 2002 the favored destination countries of a sample of Registered Nurses in South Africa who had just finished their training if they were to work outside South Africa [14]. Of these, 24% mentioned countries outside Africa not included in the nine considered here: Ireland (2%), New Zealand (4%), and Saudi Arabia (18%). An additional 11% mentioned unspecified "other countries in Europe and Africa", a subset of which may be included in the nine countries considered here. These results are somewhat difficult to interpret since, of the 105 people who answered the survey, only 85 stated that they would ever consider working outside the country while 91 gave a favored destination if they were to work outside the country. The 105 respondents were self-selected from a pool of 500 nurses initially contacted, so nonresponse bias in these numbers is a real possibility. Note also that direct recruitment of nurses by Saudi Arabia in South Africa is a very recent phenomenon, meaning that the proportion of emigrating South African Registered Nurses who went to Saudi Arabia before the year 2000 is certainly much lower than 18%.

Both in the surveys of Awases et al. and of Oosthuizen a small fraction of emigrating African health professionals reveal the intent to work in another African country, a flow which is not captured by the data presented here and which represents a small discrepancy between these numbers and true emigration to all other countries. It is smaller still when one considers reciprocal flows: A small number of emigrating Nigerian physicians go to work in Ghana, but a small number of emigrating Ghanaian physicians go to work in Nigeria. Counting each as an additional loss would ignore the fact that for intra-Africa movements, one country's loss is another's gain. And this discrepancy, to the extent that it is small and largely independent of country characteristics, contributes primarily white noise to the data here rather than any bias that would affect the analysis. In sum, the true number of health professionals working abroad may exceed the number working in the nine destination countries focused on here by an amount on the order of 5–10%. There is little reason to think that this discrepancy is systematic across countries, so the indicator remains a good estimate of the relative degree of health professional emigration across countries.

## Results

**Figure 1:**
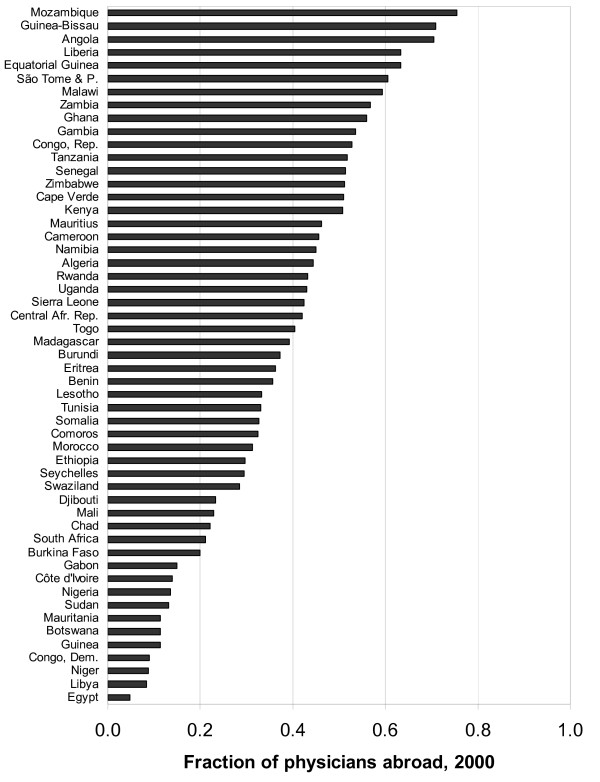
Fraction of African-born physicians residing and working abroad circa 2000.

**Figure 2:**
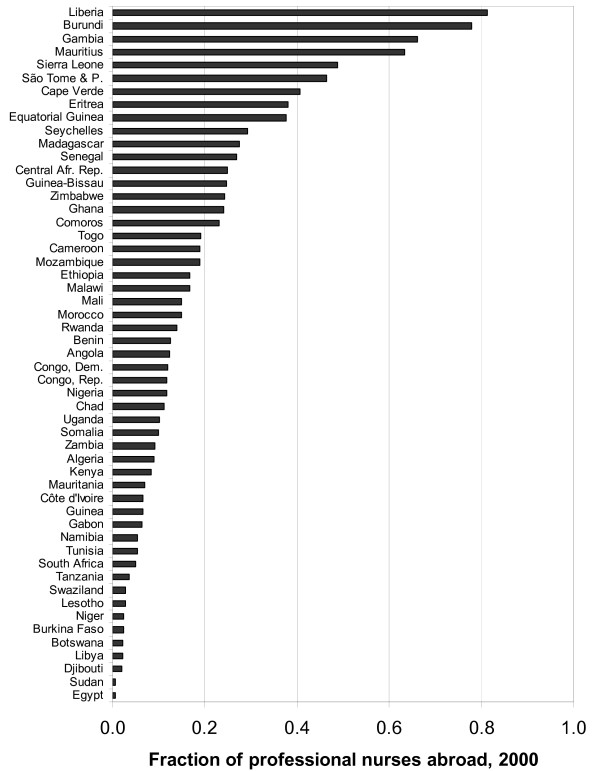
Fraction of African-born professional nurses residing and working abroad circa 2000.

Table 1 presents the number of African-born physicians residing in the nine principal destination countries circa 2000, and Table 2 presents the numbers for professional nurses. Combined with statistics for the number of physicians and professional nurses who live and work in each African country, this allows us to estimate – in the final column of each table – the fraction of total doctors and nurses born in each African country who live abroad. Figure 1 presents this fraction graphically for physicians from all African countries, and Figure 2 does the same for nurses. Note well that the numbers of doctors and nurses working *in *each African country includes those of all countries of birth.

**Table 1:** Physicians born in Africa appearing in census of nine receiving countries circa 2000

		*Receiving country*									*Total abroad*	Frac.*
				
*Sending country*	*Domestic*	GBR	USA	FRA	CAN	AUS	PRT	ESP	BEL	ZAF		
Algeria	*13,639*	45	50	10,594	10	0	2	60	99	0	*10,860*	*44%*
Angola	*881*	16	0	5	25	0	2,006	14	5	31	*2,102*	*70%*
Benin	*405*	0	4	206	0	0	0	1	13	0	*224*	*36%*
Botswana	*530*	28	10	0	0	3	0	0	1	26	*68*	*11%*
Burkina Faso	*314*	0	0	77	0	0	0	0	1	0	*78*	*20%*
Burundi	*230*	5	0	53	10	3	0	1	55	9	*136*	*37%*
Cameroon	*1,007*	49	170	332	20	0	0	4	267	3	*845*	*46%*
Cape Verde	*202*	0	15	10	0	0	186	0	0	0	*211*	*51%*
Cent. Afr. Rep.	*120*	0	0	79	0	0	2	1	5	0	*87*	*42%*
Chad	*248*	0	0	69	0	0	0	0	1	0	*70*	*22%*
Comoros	*50*	0	0	20	0	0	0	0	1	3	*24*	*32%*
Congo, DR	*5,647*	37	90	139	35	0	42	4	107	98	*552*	*9%*
Congo, Rep.	*670*	11	15	468	0	0	49	4	65	135	*747*	*53%*
Cote d'Ivoire	*1,763*	0	10	262	0	0	0	1	8	3	*284*	*14%*
Djibouti	*86*	0	0	25	0	0	0	0	1	0	*26*	*23%*
Egypt	*143,555*	1,465	3,830	471	750	535	1	17	31	19	*7,119*	*5%*
Eq. Guinea	*47*	0	0	4	0	0	1	76	0	0	*81*	*63%*
Eritrea	*173*	18	55	0	20	5	0	0	0	0	*98*	*36%*
Ethiopia	*1,310*	65	420	16	30	9	1	1	2	9	*553*	*30%*
Gabon	*368*	0	0	61	0	0	0	0	4	0	*65*	*15%*
Gambia	*40*	16	30	0	0	0	0	0	0	0	*46*	*53%*
Ghana	*1,294*	590	850	16	95	0	0	4	2	82	*1,639*	*56%*
Guinea	*898*	3	15	69	10	0	0	11	7	0	*115*	*11%*
Guinea-Bissau	*103*	0	15	75	0	0	160	0	1	0	*251*	*71%*
Kenya	*3,855*	2,733	865	0	180	110	1	4	1	81	*3,975*	*51%*
Lesotho	*114*	8	0	0	0	0	0	0	0	49	*57*	*33%*
Liberia	*73*	10	105	5	0	0	0	5	1	0	*126*	*63%*
Libya	*6,371*	349	120	20	75	5	0	9	7	0	*585*	*8%*
Madagascar	*1,428*	6	30	878	0	0	0	0	6	0	*920*	*39%*
Malawi	*200*	191	40	0	0	10	2	1	1	48	*293*	*59%*
Mali	*529*	0	15	138	0	0	0	0	4	0	*157*	*23%*
Mauritania	*333*	0	10	28	0	0	0	4	1	0	*43*	*11%*
Mauritius	*960*	294	35	307	110	36	1	0	20	19	*822*	*46%*
Morocco	*14,293*	33	225	5,113	70	4	9	833	213	6	*6,506*	*31%*
Mozambique	*435*	16	20	0	10	3	1,218	4	2	61	*1,334*	*75%*
Namibia	*466*	37	15	0	30	9	0	0	0	291	*382*	*45%*
Niger	*386*	0	10	23	0	0	0	1	3	0	*37*	*9%*
Nigeria	*30,885*	1,997	2,510	29	120	0	1	13	6	180	*4,856*	*14%*
Rwanda	*155*	4	25	8	0	0	1	0	70	10	*118*	*43%*
Sao Tome & P.	*63*	0	0	0	0	0	96	1	0	0	*97*	*61%*
Senegal	*640*	0	40	603	10	0	1	9	12	3	*678*	*51%*
Seychelles	*120*	29	0	4	10	3	0	0	0	4	*50*	*29%*
Sierra Leone	*338*	118	115	9	0	0	0	0	3	4	*249*	*42%*
Somalia	*310*	53	70	0	25	3	0	0	0	0	*151*	*33%*
South Africa	*27,551*	3,509	1,950	16	1,545	1,111	61	5	0	-834**	*7,363*	*21%*
Sudan	*4,973*	606	65	17	15	40	0	1	4	10	*758*	*13%*
Swaziland	*133*	4	4	0	0	0	1	0	0	44	*53*	*28%*
Tanzania	*1,264*	743	270	4	240	54	1	1	3	40	*1,356*	*52%*
Togo	*265*	0	10	168	0	0	0	0	2	0	*180*	*40%*
Tunisia	*6,459*	16	30	3,072	10	0	0	4	60	0	*3,192*	*33%*
Uganda	*2,429*	1,136	290	1	165	61	1	1	3	179	*1,837*	*43%*
Zambia	*670*	465	130	0	40	39	3	0	3	203	*883*	*57%*
Zimbabwe	*1,530*	553	235	0	55	97	12	1	6	643	*1,602*	*51%*
												
Africa	*280,808*	15,258	12,813	23,494	3,715	2,140	3,859	1,096	1,107	1,459	*64,941*	*19%*
Sub-Saharan	*96,405*	13,350	8,558	4,199	2,800	1,596	3,847	173	696	1,434	*36,653*	*28%*

Sources: See Acknowledgements. African sending countries show country of birth as recorded in the receiving-country census. Receiving countries show country of residence at the time of the last census (France [FRA] 1999; United States [USA] 2000; Australia [AUS], Belgium [BEL], Canada [CAN], Portugal [PRT], South Africa [ZAF], Spain [ESP], and United Kingdom [GBR] 2001). The copyright to some of the data in this table is retained by the source agency; see appendix for details before reproducing these data elsewhere. All data used here with written permission. *Gives the number of professionals abroad as a fraction of total professionals (domestic + abroad). **There are 834 physicians born in one of the other eight receiving countries who appear in the 2001 census of South Africa. This negative number thus represents a "netting out" term.

The full contents of this table are available in an Excel workbook from the Center for Global Development website [17].

**Table 2:** Professional nurses born in Africa appearing in census of nine receiving countries circa 2000

		*Receiving country*									*Total abroad*	Frac.*
				
*Sending country*	*Domestic*	GBR	USA	FRA	CAN	AUS	PRT	ESP	BEL	ZAF		
Algeria	83,022	37	138	7,953	40	6	1	26	44	0	8,245	9%
Angola	13,135	22	135	12	10	4	1,639	8	11	0	1,841	12%
Benin	1,315	4	28	155	0	0	0	0	0	0	187	12%
Botswana	3,556	47	28	0	0	0	0	0	0	5	80	2%
Burkina Faso	3,097	0	14	50	0	0	0	1	11	0	76	2%
Burundi	38	10	14	1	25	0	0	0	83	0	134	78%
Cameroon	4,998	118	664	343	0	0	0	5	33	0	1,163	19%
Cape Verde	355	0	91	25	0	0	128	0	0	0	244	41%
Cent. Afr. Rep.	300	3	6	85	0	0	0	0	6	0	99	25%
Chad	1,054	0	21	110	0	0	0	0	0	0	131	11%
Comoros	231	0	6	64	0	0	0	0	0	0	70	23%
Congo, DR	16,969	44	207	206	50	0	9	4	1,761	7	2,288	12%
Congo, Rep.	4,933	28	114	369	0	0	14	4	122	9	660	12%
Cote d'Ivoire	7,233	0	185	302	0	0	0	0	22	0	509	7%
Djibouti	424	0	0	9	0	0	0	0	0	0	9	2%
Egypt	187,017	108	661	89	45	87	0	2	0	0	992	1%
Eq. Guinea	162	0	0	0	0	0	0	98	0	0	98	38%
Eritrea	811	27	384	0	75	11	0	0	0	0	497	38%
Ethiopia	5,342	61	888	16	75	37	0	0	0	0	1,077	17%
Gabon	1,554	0	14	93	0	0	0	0	0	0	107	6%
Gambia	144	57	221	4	0	0	0	0	0	0	282	66%
Ghana	14,972	2,381	2,101	1	275	0	0	2	0	6	4,766	24%
Guinea	3,847	0	171	53	10	0	0	27	6	0	267	6%
Guinea-Bissau	799	5	0	45	0	0	212	0	0	0	262	25%
Kenya	26,267	1,336	765	4	135	110	0	0	0	22	2,372	8%
Lesotho	1,266	5	6	0	0	0	0	0	0	25	36	3%
Liberia	185	28	773	5	0	0	0	1	0	0	807	81%
Libya	17,779	72	299	1	10	7	0	2	0	0	391	2%
Madagascar	3,088	4	43	1,096	10	0	1	1	17	0	1,171	28%
Malawi	1,871	171	171	0	10	14	0	0	0	11	377	17%
Mali	1,501	0	57	208	0	0	0	0	0	0	265	15%
Mauritania	1,580	0	21	94	0	0	0	2	0	0	117	7%
Mauritius	2,629	4,042	107	86	75	195	1	0	22	3	4,531	63%
Morocco	29,462	47	276	3,707	60	4	5	560	517	0	5,176	15%
Mozambique	3,664	12	64	0	10	0	748	2	6	11	853	19%
Namibia	2,654	18	6	0	0	4	1	0	6	118	152	5%
Niger	2,668	0	28	38	0	0	0	0	0	0	66	2%
Nigeria	94,747	3,415	8,954	24	160	0	0	8	6	12	12,579	12%
Rwanda	1,805	13	85	24	20	3	1	1	144	0	292	14%
Sao Tome & P.	172	0	0	8	0	0	141	0	0	0	149	46%
Senegal	1,887	3	102	584	0	0	0	0	6	0	695	27%
Seychelles	422	80	28	8	30	29	0	0	0	0	175	29%
Sierra Leone	1,524	747	696	4	10	0	0	0	0	0	1,457	49%
Somalia	1,486	76	47	8	30	3	0	0	0	0	164	10%
South Africa	90,986	2,884	877	20	275	955	58	3	33	-261**	4,844	5%
Sudan	26,730	42	85	12	20	7	0	0	0	0	166	1%
Swaziland	3,345	21	36	0	10	4	0	0	0	25	96	3%
Tanzania	26,023	446	228	0	240	32	2	1	0	4	953	4%
Togo	782	10	36	140	0	0	0	0	0	0	186	19%
Tunisia	26,389	11	64	1,365	20	0	0	1	17	0	1,478	5%
Uganda	9,851	714	291	0	75	29	0	1	0	12	1,122	10%
Zambia	10,987	664	299	0	25	68	2	0	0	52	1,110	9%
Zimbabwe	11,640	2,834	440	0	35	219	14	3	0	178	3,723	24%
												
Africa	758,698	20,647	20,983	17,421	1,865	1,828	2,977	763	2,872	239	69,589	8%
Sub-Saharan	414,605	20,372	19,545	4,297	1,690	1,724	2,971	172	2,294	239	53,298	11%

Sources: See Acknowledgements. African sending countries show country of birth as recorded in the receiving-country census. Receiving countries show country of residence at the time of the last census (France [FRA] 1999; United States [USA] 2000; Australia [AUS], Belgium [BEL], Canada [CAN], Portugal [PRT], South Africa [ZAF], Spain [ESP], and United Kingdom [GBR] 2001). The copyright to some of the data in this table is retained by the source agency; see appendix for details before reproducing these data elsewhere. All data used here with written permission. *Gives the number of professionals abroad as a fraction of total professionals (domestic + abroad). **There are 261 professional nurses born in one of the other eight receiving countries who appear in the 2001 census of South Africa. This negative number thus represents a "netting out" term.

The full contents of this table are available in an Excel workbook from the Center for Global Development website [17].

Approximately 65,000 African-born physicians and 70,000 African-born professional nurses were working overseas in a developed country in the year 2000. This represents about one fifth of African-born physicians in the world, and about one tenth of African-born professional nurses. The fraction of health professionals abroad varies enormously across African countries, from 1% to over 70% according to the occupation and country.

## Discussion

The purpose of this note is to describe and disseminate the data rather than engage in extensive analysis. Several features of the data nevertheless leap out of the figures. The first is the extreme size of the health professional diaspora, for some countries, relative to the domestic workforce. For every Liberian physician working in Liberia, about two live abroad in developed countries; for every Gambian professional nurse working in the Gambia, likewise about two live in a developed country overseas. Also notable in the figures is the extreme variation of these statistics across the continent; Niger has a tiny physician diaspora; Ghana's is enormous.

Figure 1 also suggests a relationship between the loss of professionals and economic and political stability. Angola, Congo-Brazzaville, Guinea-Bissau, Liberia, Mozambique, Rwanda, and Sierra Leone all experienced civil war in the 1990s and all had lost more than 40% of their physicians by 2000. Kenya, Tanzania, and Zimbabwe all experienced decades of economic stagnation in the late 20th century and by its end, each had lost more than half of its physicians. Countries with greater stability and prosperity – Botswana, South Africa, and pre-collapse Côte d'Ivoire – managed to keep their doctors. It further appears that physicians may not leave countries too destitute to educate large numbers of doctors with the financial capital or connections abroad that facilitate emigration – such as Congo-Kinshasa, Niger, and pre-pipeline Chad. All three of these are among the poorest countries on earth, are not the site of any of Africa's strongest medical schools, and have very low physician emigration rates. Large countries (Nigeria, South Africa) appear better at generating domestic opportunities for health professionals. Doctors from Francophone African countries may face language barriers or other impediments in the destination countries with the most opportunities for foreign doctors. These are simple correlations; establishing causal relationships awaits more systematic analysis of these numbers.

It is important to point out that most publicly released custom tabulations from census data either contain small random perturbations or are scaled up from a random sample of the full census database using sampling weights, both of which seek to protect the privacy of individual census respondents. While the size of these alterations makes them immaterial to the analysis in this paper, it should be borne in mind that 1) the numbers in Tables 1 and 2 are not an exact representation of the full census results and 2) a separately-prepared custom extract of precisely the same variables from the same census may yield slightly different numbers.

## Conclusion

Researchers performing quantitative analysis of the effects of international trade on development can purchase detailed bilateral trade statistics from the International Monetary Fund, disaggregated by product and service with great detail. Those studying international investment flows have ready access to bilateral data from the World Bank and the United Nations disaggregated by financial instrument. But there exists no comprehensive and systematic bilateral database of the international flows of people for all countries, much less one that provides details about the migrants such as their occupation. All developed countries collect occupation-specific data on people who arrive in the country but most do not do so for people who depart the country, making high-frequency occupation-specific data on bilateral gross migration flows impossible to compile.

Until such a database exists, quantitative study of this crucial aspect of globalization will be impeded. Researchers will face the labor-intensive task of compiling data anew for each investigation. We are currently using the numbers reported here in concert with other data to perform the first systematic quantitative analysis of the effects of health professional emigration on health system staffing and health care availability in Africa, the first systematic calculation of return-migration rates for African professionals, and the first systematic calculation of the net fiscal impact of African health professional emigration. These are the first papers in a large-scale research initiative on the effects of developed-country immigration policy on poor countries.

## Competing interests

The author(s) declare that they have no competing interests.

## Authors' contributions

MC conceived of the study; MC and GP worked with census bureaus and other statistical agencies to compile and standardize the data. Both authors read and approved the final manuscript.
